# *Lonicera caerulea* L. polyphenols improve short-chain fatty acid levels by reshaping the microbial structure of fermented feces *in vitro*

**DOI:** 10.3389/fmicb.2023.1228700

**Published:** 2023-10-27

**Authors:** Xinbo Cao, Xuemeng Wang, Yanxin Ren, Yangcun Sun, Zhichao Yang, Jingping Ge, Wenxiang Ping

**Affiliations:** ^1^Engineering Research Center of Agricultural Microbiology Technology, Ministry of Education and Heilongjiang Provincial Key Laboratory of Plant Genetic Engineering and Biological Fermentation Engineering for Cold Region and Key Laboratory of Microbiology, College of Heilongjiang Province and School of Life Sciences, Heilongjiang University, Harbin, China; ^2^Hebei Key Laboratory of Agroecological Safety, Hebei University of Environmental Engineering, Qinhuangdao, China

**Keywords:** type 2 diabetes, *Lonicera caerulea* L. polyphenols, SCFAs, intestinal microbiota, fermented polyphenols

## Abstract

Increasing evidence suggests that the pathogenesis of type 2 diabetes mellitus (T2DM) is closely related to the gut microbiota. Polyphenols have been shown to alleviate T2DM, but the effects of *L. caerulea* L. polyphenols (LPs) on the gut microbiota and metabolites remain elusive. In this study, the inhibitory effects of fermented *L. caerulea* L. polyphenols (FLPs) and unfermented *L. caerulea* L. polyphenols (ULPs) on α-amylase and α-glucosidase and the impact of LP on the gut microbiota and metabolites were investigated. Furthermore, the relationship between the two was revealed through correlation analysis. The results showed that ULP and FLP had the highest inhibitory rates against α-amylase and α-glucosidase at 4 mg ml^−1^, indicating a strong inhibitory ability. In addition, LP plays a regulatory role in the concentration of short-chain fatty acids (SCFAs) and tends to restore them to their normal levels. LP reversed the dysbiosis of the gut microbiota caused by T2DM, as evidenced by an increase in the abundance of bacterial genera such as *Lactobacillus*, *Blautia*, and *Bacteroides* and a decrease in the abundance of bacterial genera such as *Escherichia-Shigella* and *Streptococcus*. Similarly, after LP intervention, the relationships among microbial species became more complex and interconnected. In addition, the correlation between the gut microbiota and metabolites was established through correlation analysis. These further findings clarify the mechanism of action of LP against T2DM and provide a new target for T2DM interventions.

## 1. Introduction

Type 2 diabetes mellitus (T2DM) is a chronic disease characterized by inflammation and is commonly referred to as non-insulin-dependent diabetes (Wang et al., 2020). The hallmark of T2DM is elevated blood glucose levels and insulin resistance, accompanied by insufficient insulin secretion and dyslipidaemia (Kahn et al., 2006; Kubota et al., 2017). According to the International Diabetes Federation report, there are currently 463 million adults worldwide with diabetes, 90% of whom have T2DM. It is estimated that this number will reach 700 million by 2045 (Xia et al., 2022). The structural and functional damage of pancreatic β-cells is often attributed to the release of inflammatory factors. In severe cases, this can even cause insulin secretion dysfunction and transport function impairment (Yang et al., 2019). Currently, the conventional treatment options for T2DM mainly involve stimulating insulin secretion, reducing glucose production, and enhancing the effect of insulin on target tissues, thereby reducing blood glucose levels (Chen et al., 2022; Zhou et al., 2023).

In recent years, many studies have found that the intestinal microbiota (IM) is also a factor involved in diabetes treatment and is associated with inflammation, insulin resistance, and pancreatic function (Salamone et al., 2021). Due to its sensitive response to various factors, it is considered an important and valuable tool for the development and promotion of human health (Yang et al., 2020). Changes in the composition of the IM can trigger inflammation, which in turn leads to changes in various gut-heart and gut-liver axis indicators (Halim and Halim, 2019). *Faecalibacterium*, *Roseburia*, and *Bifidobacterium* can significantly reduce gut permeability (Cani et al., 2009), and a decrease in their abundance can induce inflammation, increase gut permeability, and upregulate the content of lipopolysaccharide (LPS) in circulation, further disrupting glucose metabolism and insulin signaling pathways (Sedighi et al., 2017). Thus, the steady state of glucose metabolism is disrupted, leading to T2DM.

Acetic acid, propionic acid, and butyric acid account for 90–95% of the total short-chain fatty acid (SCFAs) (Koh et al., 2016); SCFAs are primarily produced by the microbial fermentation of carbohydrates (Canfora et al., 2019). SCFAs are the main metabolic products of the IM involved in the response to carbohydrate utilization (Federici, 2019), and their types and concentrations are influenced by microbial species. For example, Bacteroidetes are producers of acetate and propionate, and Firmicutes can produce butyrate (Alexander et al., 2019). The level of SCFAs is an indicator of immune, inflammatory, and metabolic characteristics. SCFAs can lower the pH in the lumen, regulate glucose metabolism, and alleviate symptoms of T2DM (Kappel and Federici, 2019). IM imbalance can reduce SCFA levels (Xu et al., 2022). Under external stimuli, changes in the composition of the gut microbiota can regulate SCFA concentration and type.

Lipopolysaccharide (LPS) is an endotoxin secreted by gram-negative bacteria in the gut. LPS promotes the secretion of proinflammatory cytokines (TNF-α and IL-6) by monocytes and macrophages, leading to chronic inflammatory reactions (Liang et al., 2013). It also inhibits the phosphorylation of insulin receptors, resulting in reduced insulin sensitivity and insulin resistance, which are the main causes of T2DM.

Chronic postprandial hyperglycaemia is a characteristic of insulin resistance and can lead to dyslipidaemia and inflammatory gene expression (Rahimi-Madiseh et al., 2017). Postprandial hyperglycaemia occurs after high carbohydrate intake and can be prevented by hydrolysis of starch by α-amylase and α-glucosidase and glucose absorption in the small intestine (Taslimi et al., 2018). Currently, commercially available hypoglycaemic drugs are sufficient for the treatment of diabetes but have drawbacks such as adverse reactions and drug resistance (Venkatakrishnan et al., 2019). Polyphenols are a new type of natural hypoglycaemic compound that can lower blood sugar levels by inhibiting α-amylase and α-glucosidase in the small intestine (Pradeep and Sreerama, 2015).

Polyphenols are the seventh nutrient for humans and have the advantages of wide availability and abundant resources (Caprioli et al., 2016). Recently, it has been found that polyphenols play an outstanding role in disease treatment. For example, grape polyphenols can regulate the gut microbiota and increase the mRNA expression of key intestinal barrier genes (Lu et al., 2021). Curcumin has a significant therapeutic effect in alleviating high-fat diet-induced hepatic steatosis and obesity (Li et al., 2021a). Therefore, the use of polyphenols is considered a new therapeutic strategy for various metabolic diseases, such as T2DM, as they can regulate the gut microbiota and improve the inflammatory response (Son et al., 2019).

*Lonicera caerulea* L., also known as blue honeysuckle, is a small berry tree species that grows in the northern frigid zone. It is rich in nutrients, such as amino acids and vitamins, especially polyphenols, which are important active substances in *L. caerulea* L. (Sharma and Lee, 2021). However, there is still limited research on the development and functional verification of *Lonicera caerulea* L. polyphenols (LPs). This is partly due to the limited availability of *L. caerulea* L. resources and the low utilization rate of plant polyphenols. In particular, in *L. caerulea* L. cells, polyphenols exist in both free and bound forms. Additionally, conventional physical and chemical extraction methods have low yields and may leave chemical residues (de Araujo et al., 2021). Recent studies have found that LP can reduce oxidative stress damage by affecting lipid synthesis and has certain value in reducing fatigue (Golba et al., 2020; Dayar et al., 2021).

In recent years, *in vitro* fecal fermentation technology has been an effective tool for screening large amounts of substrates, including dietary components, drugs, and toxic or radioactive components. It can also determine changes in the gut microbial environment (Wang et al., 2019). The main purpose of fecal fermentation models is to cultivate complex gut microbial communities under controlled conditions for research related to microbiota regulation and metabolism (Nishikawa et al., 2009). Compared to animal experiments, *in vitro* fermentation experiments are more economical and allow easier control of experimental parameters. They can also be repeated multiple times, making them an important tool for studying the interactions between different compounds and the gut microbiota, and they can serve as preliminary experiments for animal studies (Jonathan et al., 2012). Cheng used *L. caerulea* L. pomace polyphenols for fecal fermentation; these compounds improved the microbial community structure, increased short-chain fatty acid production, and served as a predictor of gut health in subsequent *in vivo* studies in mice (Cheng et al., 2020). Additionally, apple peel polyphenols have also shown potential benefits for gut health when used in fecal fermentation, but the health-promoting effects of polyphenols *in vivo* need further research (Zahid et al., 2023).

Therefore, in this study, we hypothesized that using microbial fermentation can increase the extraction efficiency of LP. Fermented LP can reshape the microbial community composition and diversity in *in vitro* fecal fermentation, increase the content of SCFAs that maintain gut homeostasis, and reduce the content of LPS, which causes inflammatory reactions, making it an effective tool for preventing T2DM. The aims of this study were to (1) analyze the effect of fermented LP on key enzyme activities associated with T2DM and evaluate the possibility of using fermented LP to prevent T2DM, (2) establish an *in vitro* fecal fermentation system and reveal the effect of fermented LP on the microbial community structure from mouse feces, (3) explore the effect of fermented LP on SCFA and LPS contents, (4) explore the core microbial community for SCFA and LPS production, and (5) elucidate the mechanisms of the microbial metabolism pathway mediated by fermented LP. The results of this study will provide new insights into the mechanisms of action of LP in the mouse colon environment and provide a theoretical basis for further research on the effects of LP on T2DM.

## 2. Materials and methods

### 2.1. Preparation of LP

*Lonicera caerulea* L. was provided by Professor Junwei Huo of Northeast Agricultural University and Harbin Senmeiyuan Biotechnology Co., Ltd., and the variety is named Lanjingling. The *Lactobacillus rhamnosus* CICC6224 strain was purchased from the China Center of Industrial Culture Collection.

The LPs were divided into unfermented polyphenols (ULPs) and fermented polyphenols (FLPs). FLP was a homogenate obtained by crushing *L. caerulea* L. in deionized water at a ratio of 1:2.5, inoculating with 3.5% *L. rhamnosus* 6,224 (10^8^ CFU mL^−1^) and fermenting for 28 h before extraction. ULPs were directly extracted by crushing *L. caerulea* L. in deionized water at a ratio of 1:2.5. The extraction method for ULPs and FLPs was improved according to Savikin (Šavikin et al., 2021). The fermentation broth or the homogenate of *L. caerulea* L. was added to a 60% ethanol solution and ultrasonically extracted at 50°C for 90 min with 550 W power (Wang R. et al., 2022; Wang S. et al., 2022). After extraction, it was placed into an ice box for quick cooling and centrifuged at 12000 r min^−1^ for 15 min to obtain the supernatant, which was further evaporated by vacuum rotation at 50°C to harvest crude polyphenols. AB-8 macroporous adsorption resin was pretreated and used to obtain purified polyphenols with the following steps: soaking in 95% ethanol for 24 h, washing in distilled water to neutral pH, soaking again in 5% sodium hydroxide solution for 5 h, and finally washing with distilled water to neutral pH (Zhang J. et al., 2022; Zhang S. S. et al., 2022). The treated AB-8 resin was placed in distilled water. A column (1.6 cm × 50 cm) was loaded with pretreated AB-8 by the wet method and allowed to settle naturally. After elution with deionized water for 12 h, crude polyphenols were added and loaded at a rate of 1 mL min^−1^ throughout (Shi et al., 2021). The harvested pure polyphenols were freeze-dried and were then ready for use. All of the reagents were supplied by Beijing Solarbio Technology Co., Ltd. (Beijing, China). Standards for mixed SCFAs and acarbose were purchased from Yuanye Biotechnology Co., Ltd. (Shanghai, China).

### 2.2. Inhibitory effect of LP on α-amylase and α-glucosidase activities

First, 1 mL of α-amylase solution (0.6 mg mL^−1^), 1 mL of ULP and FLP at different concentrations (0, 0.1, 0.2, 0.4, 0.6, 0.8, 1.0, 2.0, 4.0 mg mL^−1^) and 1 mL of soluble starch (2.0 mg mL^−1^) were mixed together and incubated for 20 min at 37°C. Then, 1 mL of DNS reagent was added, and the sample was heated for 5 min. This was followed by the addition of 10 mL of distilled water after cooling (Değirmencioğlu et al., 2021). The absorbance was determined at 540 nm, and acarbose was used as positive control.


$$\alpha -\mathrm{amylase inhibition rate}\left(\%\right)=\left(1-\frac{A1-A2}{A3-A4}\right)\times 100$$

A1 is the absorbance value of the sample. A2 is the absorbance value of the sample with PBS instead of α-amylase. A3 is the absorbance value of the sample with PBS instead of the sample. A4 is the absorbance value of the sample with PBS instead of the polyphenols and α-amylase.

Forty microlitres of ULP and FLP at different concentrations (0, 0.1, 0.2, 0.4, 0.6, 0.8, 1.0, 2.0, 4.0 mg mL^−1^) were mixed with 40 μL of glucosidase (1 U mL^−1^) and thoroughly shaken for 1 min. After warming at 37°C for 10 min, 20 μL of PNPG (2.5 mmol L^−1^) was added, and the sample was shaken for 1 min. At the end of the shaking, 50 μL of Na_2_CO_3_ (0.2 mol L^−1^) was added to terminate the reaction, and the reaction was allowed to proceed for 30 min (Wang R. et al., 2022; Wang S. et al., 2022). The absorbance was measured at 405 nm, and acarbose was used as the positive control.


$$\alpha -\mathrm{glucosidase inhibition rate}\left(\%\right)=\left(1-\frac{A1-A2}{A3-A4}\right)\times 100$$

A1 is the absorbance value of the sample. A2 is the absorbance value of the sample with phosphate buffer instead of PNPG. A3 is the absorbance value of the sample with phosphate buffer instead of the polyphenols. A4 is the absorbance value of the sample with phosphate buffer instead of the polyphenols and PNPG.

### 2.3. *In vitro* fecal fermentation test

#### 2.3.1. Establishment of the fermentation model *in vitro*

The feces of high-glucose mice (C57BL/6 N) were purchased from Honfer White Rat Breeding Export Base (Tsingtao China) to simulate the intestinal environment of animals (Fan J. et al., 2022; Fan Y. et al., 2022). Samples of 2 g of clean feces from 5 high-sugar obese mice (blood sugar content >300 mg 100 mL^−1^) and 2 g of clean feces from 5 normal mice were collected and immediately transferred to sterile centrifuge tubes and stored at −80°C for future use within 2 h (Lu et al., 2023). The fecal samples used in this study were obtained from 7-week-old male mice.

#### 2.3.2. Method and grouping of fecal fermentation *in vitro*

The feces of high-sugar mice or normal mice were mixed with phosphate buffer (pH 7.4) at a ratio of 1:6. The samples were homogenized to obtain a pretreated fecal slurry (Pfs), and most of the solid particles were filtered by four layers of sterilized cotton gauze. The fecal treatment liquid (Ftl) was obtained by mixing the Pfs with the reducing solution (2.52 g L-cysteine hydrochloride, 16 mL 1 mol L^−1^ NaOH, 2.56 g anhydrous sodium sulfide, 380 mL ddH_2_O) at a ratio of 1:5. Then, 27 mL of *in vitro* fermentation medium (8.0 g L^−1^ starch, 3.0 g L^−1^ peptone, 3.0 g L^−1^ tryptone, 4.5 g L^−1^ yeast extract powder, 0.4 g L^−1^ bile salt, 0.8 g L^−1^ cysteine hydrochloride, 0.05 g L^−1^ hemin, 4.5 g L^−1^ NaCl, 2.5 g L^−1^ KCl, 0.45 g L^−1^ MgCl_2_•6H_2_O, 0.20 g L^−1^ CaCl_2_•6H_2_O, 0.40 g L^−1^ KH_2_PO_4_, 1.0 mL L^−1^ Tween-80, 2.0 mL L^−1^ trace element solution) (Li et al., 2021b), 3 mL of Ftl and 2 mL of ULP/FLP were mixed and incubated in an anaerobic incubator (YQX-II) at 35°C for 48 h in triplicate. Samples were taken at 0, 6, 12, 24, and 48 h, respectively (Liu et al., 2020).

The *in vitro* fecal fermentation experiment was set up with four treatments: feces of high-glucose obese mice with unfermented polyphenols (ULP), feces of high-glucose obese mice with polyphenols after fermentation (FLP), feces of high-glucose obese mice with ddH_2_O instead of polyphenols (NC) and feces of normal mice with ddH_2_O (CK).

### 2.4. Key metabolite detection

Two millilitres of fecal fermentation samples at 0, 6, 12, 24, and 48 h from each treatment were centrifuged at 10000 r min^−1^ for 10 min at room temperature. The supernatant was filtered for metabolite detection. The levels of SCFAs were determined by HPLC and calculated by a standard curve of acetic acid, butyric acid and propionic acid, with concentration expressed as μmol mL^−1^. The lipopolysaccharide/endotoxin (LPS) ELISA kit (Shanghai Enzyme-linked Biotechnology Co., Ltd.) was used to determine the lipopolysaccharide content in the samples following the manufacturer’s instructions.

### 2.5. 16S rRNA gene sequencing analysis

Fecal fermentation samples were obtained at 0, 6, 12, 24, and 48 h for biodiversity analysis. DNA was extracted utilizing the Power Soil DNA Isolation Kit (D5625-01, Omega Biotek, Inc., United States). The PCR primers 338F (5′-ACTCCTACGGGAGGCAGCA-3′) and 806R (5′-GGACTACHVGGGTWTCTAAT-3′) targeting the 16S rRNA gene V3-V4 region were used to investigate the bacterial community structure.

### 2.6. Statistical analysis

In this experiment, data were expressed as means ± standard deviation (SD). The SCFAs and LPS content were analyzed using multiple comparison t-tests in SPSS 26, and the data was graphically visualized using Origin 2021. NMDS analysis was employed to explore the similarity or dissimilarity of community composition among different sample groups. NMDS considered the similarity or dissimilarity data as a monotonic function of the distances between points, replacing the original data with new columns in the same order while preserving the original data’s ordinal relationships for metric multidimensional scaling analysis (Fan J. et al., 2022; Fan Y. et al., 2022). Lefse analysis, a multilevel species differential discriminant analysis, utilized non-parametric factorial Kruskal-Wallis (KW) sum-rank test to detect features with significantly different abundances. Linear discriminant analysis (LDA) was used to estimate the effect size of each component (species) abundance on the differences. Network analysis was conducted using Gephi 0.95, and Spearman correlation analysis was performed among microbial genera. Microbial genera with a correlation coefficient (*r*) greater than 0.95 and a value of *p* (*p*) less than 0.05 were used for plotting (Sun et al., 2023). The correlation analysis between core microbiota and metabolites was visualized using Cytoscape v3.8.2. Core microbiota were selected based on their correlation with SCFAs, LPS, and microbes using Spearman correlation analysis, with a selection criterion of r > 0.8 and *p* < 0.05 (Kang et al., 2023). PICRUSt 2 is utilized for predicting the functional composition of microbial communities in samples based on amplicon sequencing data. Data statistical analysis was performed using Major Biopharm Technology online report.

## 3. Results and discussion

### 3.1. The effect of LP on α-amylase and α-glucosidase

**Figure 1:**
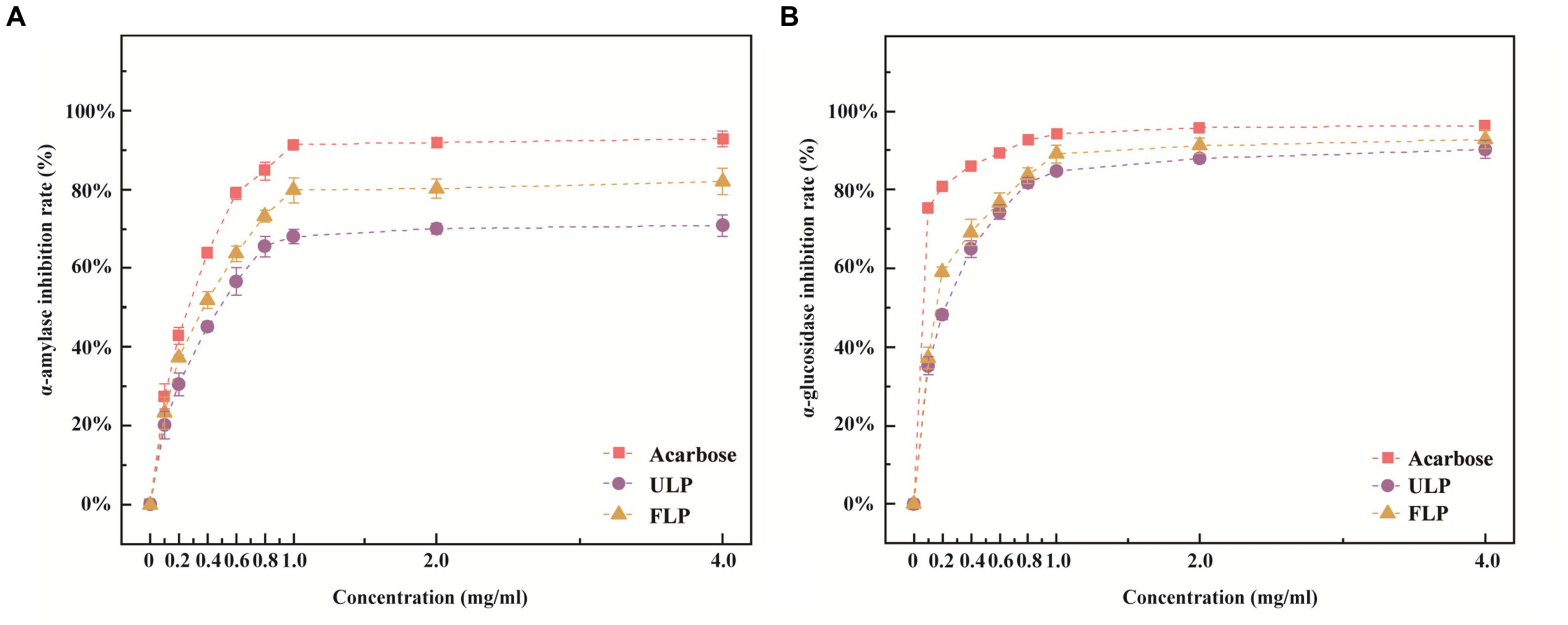
Effects of ULP and FLP on α-amylase and α-glucosidase. **(A)** Inhibition of α-amylase activity. **(B)** Inhibition of α-glucosidase activity.

First, we conducted an analysis of differentially abundant polyphenolic metabolites between ULP and FLP (Supplementary Figure S1). α-Amylase is a key enzyme that hydrolyses long carbohydrates to short glucose molecules for transport into cells. α-Amylase inhibitors block the metabolism of carbohydrates, such as that of starch to dextrin and oligosaccharides, in the body by inhibiting the activity of α-amylase in the intestine; this delays the digestion of carbohydrates in the small intestine, reduces sugar intake, and lowers postprandial blood glucose levels (Nan et al., 2022). The concentration of LP was positively correlated with the inhibition of α-amylase (Figure 1A). The inhibition of α-amylase increased from 20.17 to 70.87% when the LP concentration in the ULP group increased from 0.1 mg mL^−1^ to 4 mg mL^−1^, and the inhibition of α-amylase by LP in the FLP group increased from 23.12 to 81.98%. When the LP concentration in the ULP and FLP groups was greater than 1 mg mL^−1^, the inhibition of α-amylase by LP tended to stabilize. Compared with that of acarbose at the same concentration, the inhibitory effect of LP on α-amylase in the ULP and FLP groups was low, but the inhibitory effect in the FLP group was better than that in the ULP group (*p* < 0.05). The LP in fermented *L. caerulea* L. exerts a strong inhibitory effect on α-amylase and can be used as an α-amylase inhibitor.

α-Glucosidase is located on the brush border membrane of small intestinal cells and participates in the metabolic pathway of starch and glycogen. It hydrolyses the α-1,4-glycosidic bond at the end of oligosaccharides to release glucose, ultimately leading to hyperglycaemia. Therefore, inhibiting the activity of α-glucosidase can delay the production of glucose at the source (Valdes et al., 2020). The concentration of LP was positively correlated with the inhibitory effect of LP on α-glucosidase (Figure 1B). When the concentration of LP in the ULP group increased from 0.1 mg mL^−1^ to 4 mg mL^−1^, the α-glucosidase inhibition rate increased from 35.12 to 90.25%; in the FLP group, the value increased from 37.24 to 93.78%. When the LP concentration in the ULP and FLP groups was greater than 1 mg mL^−1^, the inhibition of α-glucosidase by LP tended to stabilize. Compared to that of acarbose at the same concentration, the inhibitory effect of LP on α-glucosidase in the ULP and FLP groups was low (*p* < 0.05). Therefore, fermented LP has a strong inhibitory effect on alpha-glucosidase and can be used as an α-glucosidase inhibitor.

### 3.2. Effects of LP on key metabolites in the fecal fermentation system *in vitro*

**Figure 2:**
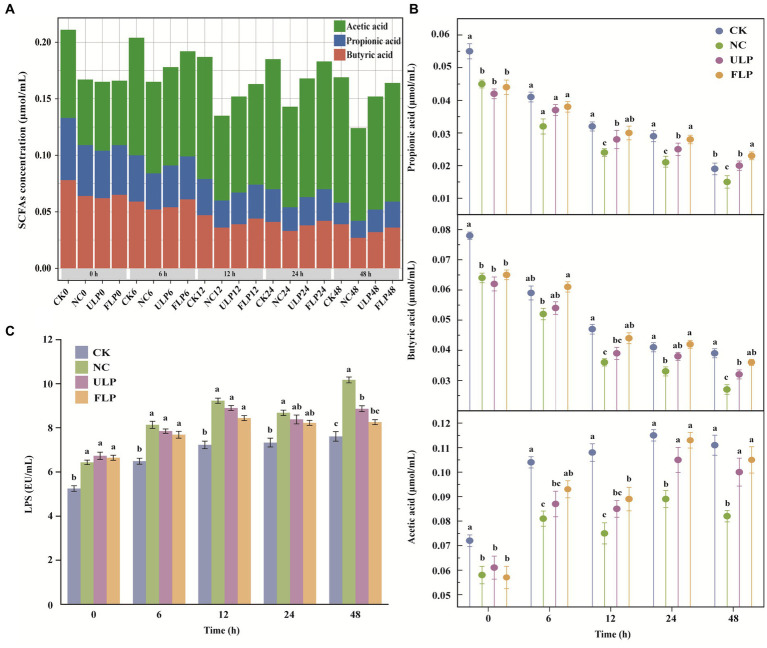
Environmental indicator detection. **(A)** Stacked chart of SCFAs. **(B)** Propionic acid, butyric acid, and acetic acid content charts. **(C)** Chart of LPS content. The statistical analysis was conducted using a *t*-test, and the error bars on the bar chart represent the standard deviation. The a-c labels on the bar chart were obtained through significance analysis using SPSS, where different letters indicate significant differences (*p* < 0.05).

Plant polyphenols can regulate the structure of the gut microbiota, promote the enrichment of SCFA-producing bacteria, and improve the content of SCFAs in the gut. Therefore, the content of SCFAs can be used as an indicator of the effect of polyphenols on the gut microbiota (Jiang et al., 2022). As fermentation progressed, the content of SCFAs in all four groups showed a decreasing trend, which might be related to the activity of the gut microbiota in the late stage of fermentation (Figure 2A). The content of acetic acid, propionic acid, and butyric acid in the NC, ULP, and FLP groups was consistently lower than that in the CK group (Figure 2B). These findings indicate that the structure of the gut microbiota in T2DM mice had changed, indirectly affecting the content of SCFAs. However, from 24 h onwards, the content of acetic acid, propionic acid, and butyric acid in the FLP group was higher than that in the ULP and NC groups. These findings indicate that the addition of polyphenols could improve the content of SCFAs in the feces of high-glucose mice, and the effect of fermented LP was superior to that of unfermented LP. LP was shown to stimulate the growth of SCFA-producing bacteria in the gut microbiota, produce beneficial SCFAs, and promote human health. Propionic acid and butyric acid were the most abundant SCFAs in all groups, and FLP had the best effect on the growth of SCFAs. Adding LP could alleviate gut microbiota disturbances in the T2DM group and bring the level of SCFA secretion from the gut closer to normal levels.

The LPS content in all four groups showed an upwards trend with the extension of fermentation time (Figure 2C), indicating that the addition of LP changed the proportion of microbial species and regulated gut microbiota disorder (Zhang J. et al., 2022; Zhang S. S. et al., 2022). Excluding the CK group, the LPS content consistently showed the order NC > ULP > FLP. This finding indicates that the addition of LP could effectively inhibit the proliferation of gram-negative bacteria, reduce the secretion of LPS, and alleviate inflammatory reactions, and the effect of fermented LP was superior to that of unfermented LP.

### 3.3. Effects of LP on microbial composition and diversity of *in vitro* fermented feces

The average sequencing depth of 60 samples from the CK, NC, ULP, and FLP groups was 428 reads, and an average of 1,144 OTUs were identified per sample based on 97% sequence similarity of high-quality sequences. α-Diversity, which reflects the biodiversity of the microbial community in *in vitro* fermented feces, can be determined by two important parameters: species evenness and richness. The Chao1 and ACE indices reflect the number of OTUs in the community, while the Shannon index is used to evaluate microbial diversity. Table 1 shows that the α-diversity of the ULP and FLP groups tended to recover toward that of the CK group, indicating the need to study T2DM from a microbial perspective.

**Table 1:** α Diversity index statistical table.

Sample	Time (h)	ACE	Chao	Simpson	Shannon	Coverage
CK	0	3.52	0.09	417.43	420.98	0.9980
	6	1.33	0.44	161.14	150.82	0.9985
	12	1.60	0.32	130.78	107.37	0.9993
	24	1.19	0.45	54.74	38.28	0.9998
	48	0.86	0.63	36.49	33.44	0.9998
NC	0	2.98	0.14	372.91	373.00	0.9981
	6	1.97	0.19	187.41	155.24	0.9986
	12	1.73	0.28	95.18	69.25	0.9996
	24	1.45	0.37	42.45	38.17	0.9998
	48	1.06	0.55	31.00	30.33	0.9999
ULP	0	3.60	0.09	473.97	473.62	0.9979
	6	2.53	0.16	273.57	269.35	0.9985
	12	1.50	0.33	101.22	66.83	0.9995
	24	1.24	0.43	68.93	45.58	0.9997
	48	0.96	0.56	87.62	70.00	0.9995
FLP	0	3.77	0.072	380.89	390.11	0.9986
	6	2.06	0.16	142.58	133.39	0.9988
	12	1.65	0.31	103.40	79.69	0.9996
	24	1.46	0.36	78.45	65.87	0.9997
	48	0.92	0.62	42.84	38.28	0.9998

**Figure 3:**
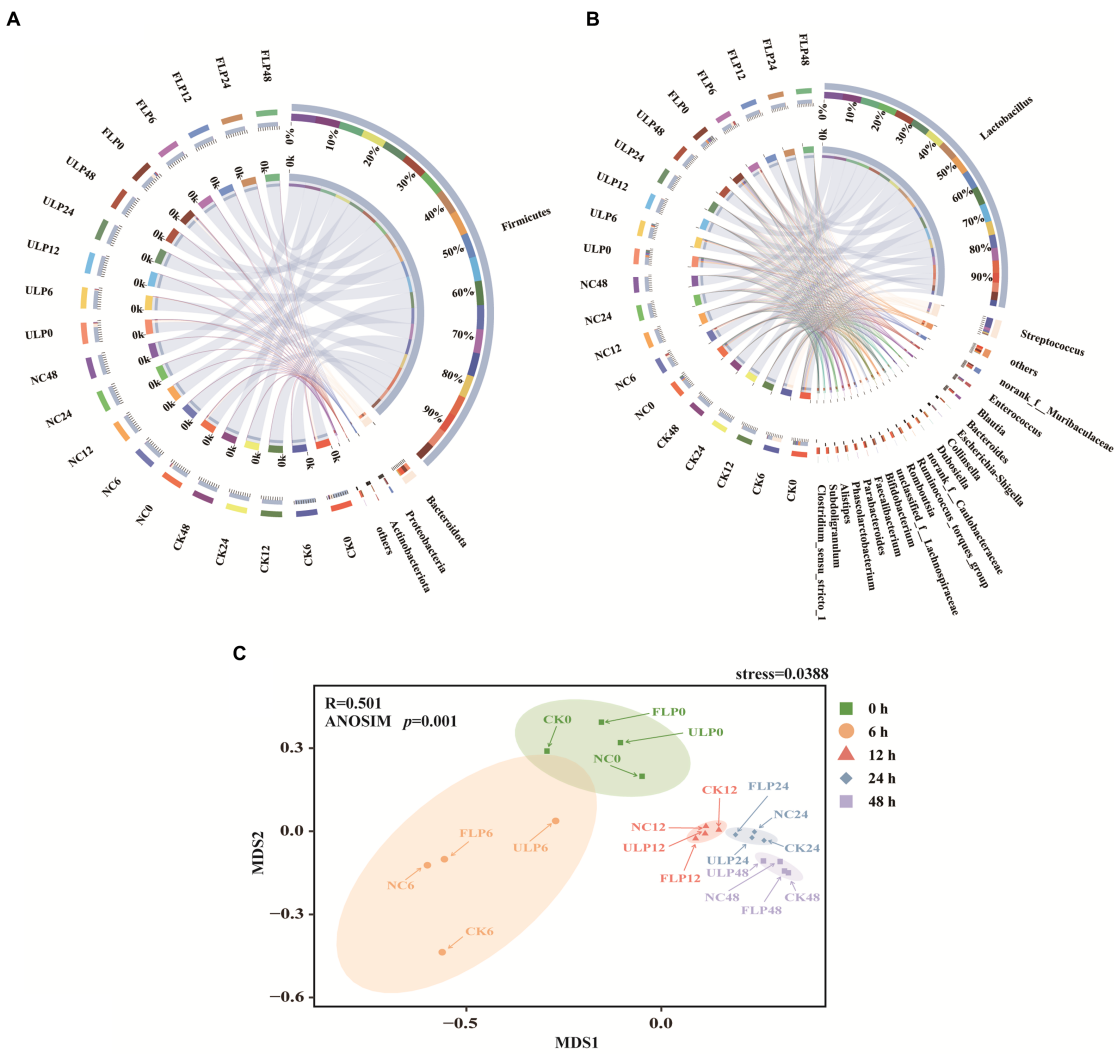
Microbial diversity analysis in the *in vitro* fecal fermentation system. **(A)** Bacterial community structure at the phylum level. **(B)** Bacterial community structure at the genus level. **(C)** NMDS analysis at different times.

At the phylum level, the microbial community structure of each group was mainly composed of four dominant phyla: Firmicutes, Bacteroidetes, Proteobacteria, and Actinobacteria (Figure 3A). These phyla accounted for 99% of the total microbial content, and Desulfobacterota was the main phylum in the remaining 1%. Throughout the fermentation process, the microbial community in *in vitro* fermented feces gradually decreased in number. Thus, Firmicutes gradually emerged as the dominant strain, as they are often better adapted to extreme environments. Some studies have shown that the ratio of Firmicutes to Bacteroidetes is related to obesity (Zheng et al., 2018). Interestingly, in this study, LP intervention reversed the F/B ratio and reduced the abundance of Firmicutes. This finding suggests that LP intervention can improve the intestinal microbiota structure (Masumoto et al., 2016).

The genera whose abundance exceeded 1% in each group included *Lactobacillus*, *Streptococcus*, *Escherichia-Shigella*, *Blautia*, *Bacteroides*, *Collinsella*, *Faecalibacterium*, *Parabacteroides*, *Subdoligranulum*, and *Phascolarctobacterium* (Figure 3B). Among these, *Lactobacillus* was the core genus, especially at 24 and 48 h, when the changes in the genera of each group tended to stabilize. *Lactobacillus* is a probiotic that colonizes the human body, and the lactic acid it produces acts as a precursor for the production of acetate (Tian et al., 2023b). At 12 h, the relative abundance of *Lactobacillus* in the CK, NC, ULP, and FLP groups increased by 22, 26, 32, and 36%, respectively, compared to that in the initial fermentation period. *Blautia* is an acetate-producing bacterium that is commonly used in the preparation of enhanced hypoglycaemic and lipid-lowering drugs (Benitez-Paez et al., 2020). At 12 h, the relative abundance of *Blautia* in the CK, ULP, FLP, and NC groups increased by 1.19-, 0.75-, 1.78-, and 2.07-fold, respectively, compared to that in the initial period, with that in ULP and FLP being 1.55 and 2.3 times higher, respectively, than that in NC. *Phascolarctobacterium* is a genus of strictly anaerobic gram-negative bacteria that can produce acetate and propionate, which regulate host metabolism and emotional changes (Ogata et al., 2019). Compared to that at 0 h, the relative abundance of *Phascolarctobacterium* in the CK group increased by 0.19-fold at 12 h. In the ULP and FLP groups, although it showed a downwards trend, the relative abundance increased by 1.18 and 2.28% compared to the NC group. *Faecalibacterium* is one of the most important bacterial genera in the human gut microbiota, and these species are important producers of butyrate and a next-generation probiotics for preventing inflammation (Zou et al., 2021). The relative abundance of *Faecalibacterium* in the CK, ULP, FLP, and NC groups increased by 1.06-, 0.45-, 0.5-, and 0.3-fold, respectively, compared to that in the initial period, with the relative abundance in ULP and FLP being 3 and 4.23 times higher, respectively, than that in NC. In this study, *Lactobacillus*, *Blautia*, *Phascolarctobacterium*, and *Faecalibacterium* have been found to be positively correlated with the production of SCFAs, these bacteria may be related to the production of SCFAs in the fecal fermentation.

*Bacteroides* are gram-negative bacteria whose outer membrane vesicles can transport virulence factors that induce inflammation. However, they can efficiently degrade carbohydrates into glucose and small sugar molecules and produce acetate, isovalerate, and succinate through anaerobic respiration (Fernandez-Julia et al., 2021). From the beginning of fermentation to 12 h, all four groups showed a decreasing trend. The abundance in the FLP group was lower than that in the ULP group, but the difference was not significant (*p* > 0.05). Although this genus can produce SCFAs, the addition of LP did not increase its abundance. *Collinsella* species produce ursodeoxycholic acid in the intestine; ursodeoxycholic acid inhibits inflammation and apoptosis and functions as an antioxidant. Recent studies have found that *Collinsella* encapsulated by nanovesicles can be used to alleviate or treat diabetes (Qin et al., 2019). The relative abundance of *Collinsella* was increased by 2.1% in the CK group at 12 h, while the NC, ULP and FLP groups showed a decreasing trend. However, the abundance in the FLP group was 1.3 times higher than that in the ULP group. *Parabacteroides* can be used as an agonist of fructose-1,6-bisphosphatase, a key enzyme of gluconeogenesis, for the treatment of insulin resistance and disorders of lipid metabolism (Wang et al., 2018). The relative abundance of *Parabacteroides* increased 0.85-fold in the 12 h CK group compared with the 0 h group. The relative abundance of *Parabacteroides* at 12 h in the ULP and FLP groups showed a decreasing trend, but the abundance in the FLP group was increased by 12% compared with that in the ULP group. Although *Collinsella* and *Parabacteroides* are beneficial to humans, their abundance was not increased by the addition of LP.

*Streptococcus* is a genus of pathogenic bacteria in the human gastrointestinal tract that often cause various inflammatory and allergic reactions, leading to a decrease in the body’s functional immunity (Li et al., 2023). Compared to that at 0 h, the relative abundance of this bacterium at 6 h in the CK, NC, ULP, and FLP groups decreased by 7, 8, 12, and 13.4%, respectively. *Escherichia-Shigella* is a genus of bacteria associated with chronic metabolic diseases that can inhibit the expression of related genes, such as *PPARα*, leading to inflammation and damage to the body (Xin et al., 2022). At 12 h, the relative abundance of this genus decreased by 4% in the CK group but increased by 4% in the NC group. Compared to the NC group, the relative abundances of this genus in the ULP and FLP groups decreased by 2.69-fold and 6.36-fold, respectively, indicating that the addition of LP can help lower the abundance of *Escherichia-Shigella* and regulate glucose and lipid metabolism in the body. *Subdoligranulum* is a genus of gram-negative bacteria found in feces, and a study found that the relative abundance of *Subdoligranulum* in T2DM patients was higher than that in the normal group (Lin et al., 2021). In this study, the relative abundance of *Subdoligranulum* in the CK group tended to approach 0 at 12 h and decreased by 1.38-fold and 2.2-fold in the ULP and FLP groups compared to the NC group, respectively. *Streptococcus*, *Escherichia-Shigella*, and *Subdoligranulum* all promote inflammation, and the addition of LP can lower their relative abundances. However, since their actual initial abundances account for only 0.7, 0.45, and 0.2% of the microbiota, respectively, the overall impact on the functional properties of fermented feces is relatively small. These results suggested that LP can serve as a potential prebiotic, regulate the microbiota structure in the intestine of T2DM patients, increase the abundance of beneficial bacteria, reduce the abundance of harmful bacteria, and maintain the stability of the intestinal environment.

Nonmetric multidimensional scaling (NMDS) is a data analysis method that simplifies the research object in a multidimensional space and locates, analyses and classifies it in a low-dimensional space while preserving the original relationships between objects. The similarity or difference of the four bacterial communities was analyzed through NMDS, as shown in Figure 3C. A stress value of 0.0388 < 0.05 indicates excellent results. The bacterial communities of the *in vitro* fecal fermentation samples were divided into five groups. There were significant differences among the communities at different fermentation times, and the communities at the same fermentation time with different treatments were closer (The *p-*value of ANOSIM test was 0.001). Shortly after fecal fermentation began, the bacterial communities showed obvious succession. The distances among each treatment within the 0 and 6 h communities were large, indicating significant differences in the bacterial composition. However, the distances among the 12, 24, and 48 h communities were short. These findings indicate that the bacterial differences among the different sample groups decreased during the later stages of fermentation, and the community structure tended to be stable. These results indicate that the addition of LP could change the microbial community structure and ultimately stabilize the structure of the fermentation samples in each group.

### 3.4. Microbial composition differences

**Figure 4:**
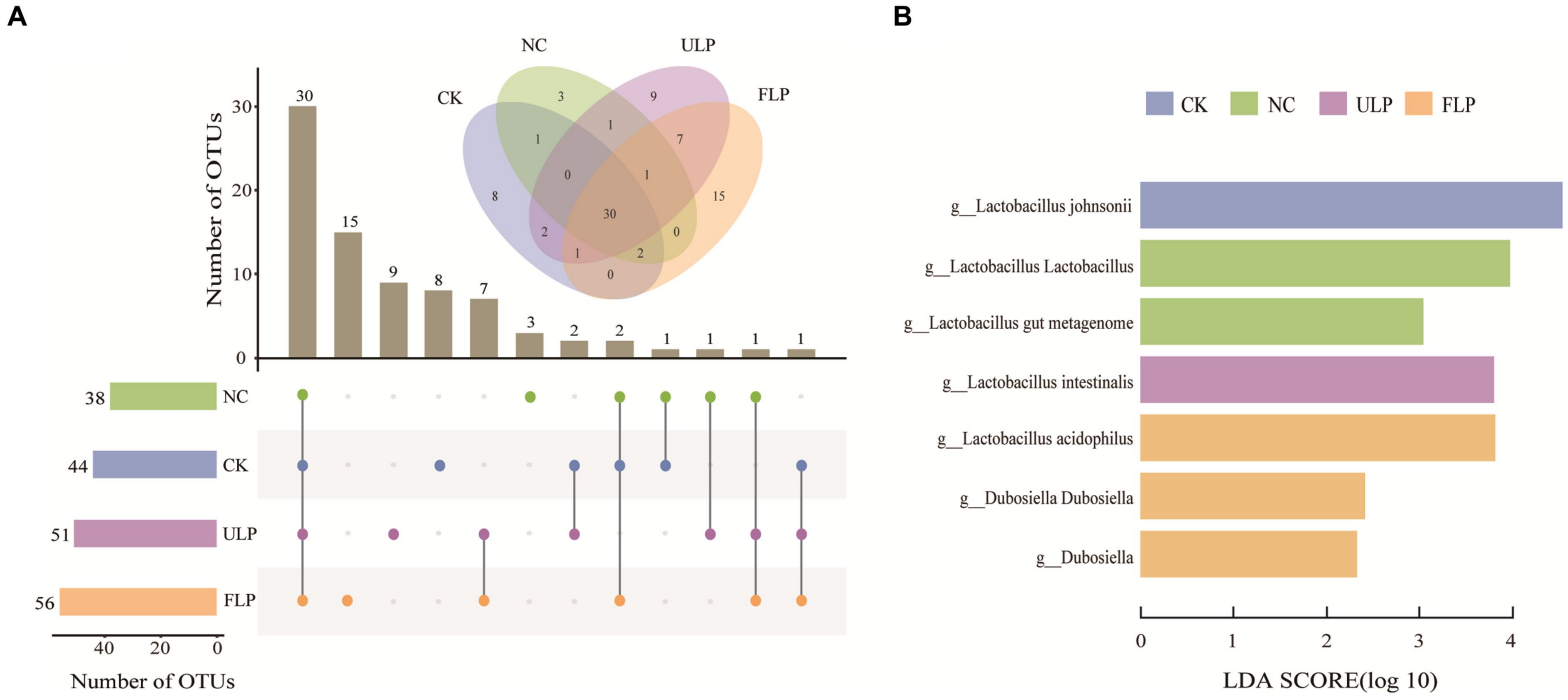
Analysis of microbial composition differences in *in vitro* fermented feces. **(A)** UpSet plot analysis at 24 h. **(B)** LDA score plot at 24 h.

An UpSet plot analysis was conducted on the community at 24 h (Figure 4A). The analysis revealed that the four treatment groups shared 30 OTUs, and the CK, NC, ULP, and FLP groups had 8, 3, 9, and 15 unique OTUs, respectively. This indicated that LP intervention could increase the number of unique bacterial genera in the FLP group. As shown Figure 4B, a higher LDA score indicated a greater effect of species abundance on the differential effect, with an LDA score > 2 indicating statistical significance. *Lactobacillus* and *Dubosiella* were the two genera with the highest LDA scores, and significant differences were observed between the groups, indicating that they can serve as biomarkers. *Dubosiella* can prevent and alleviate obesity and its complications, including hyperlipidemia, fatty liver, insulin resistance, and glucose intolerance (Liu et al., 2023).

### 3.5. Network analysis

**Figure 5:**
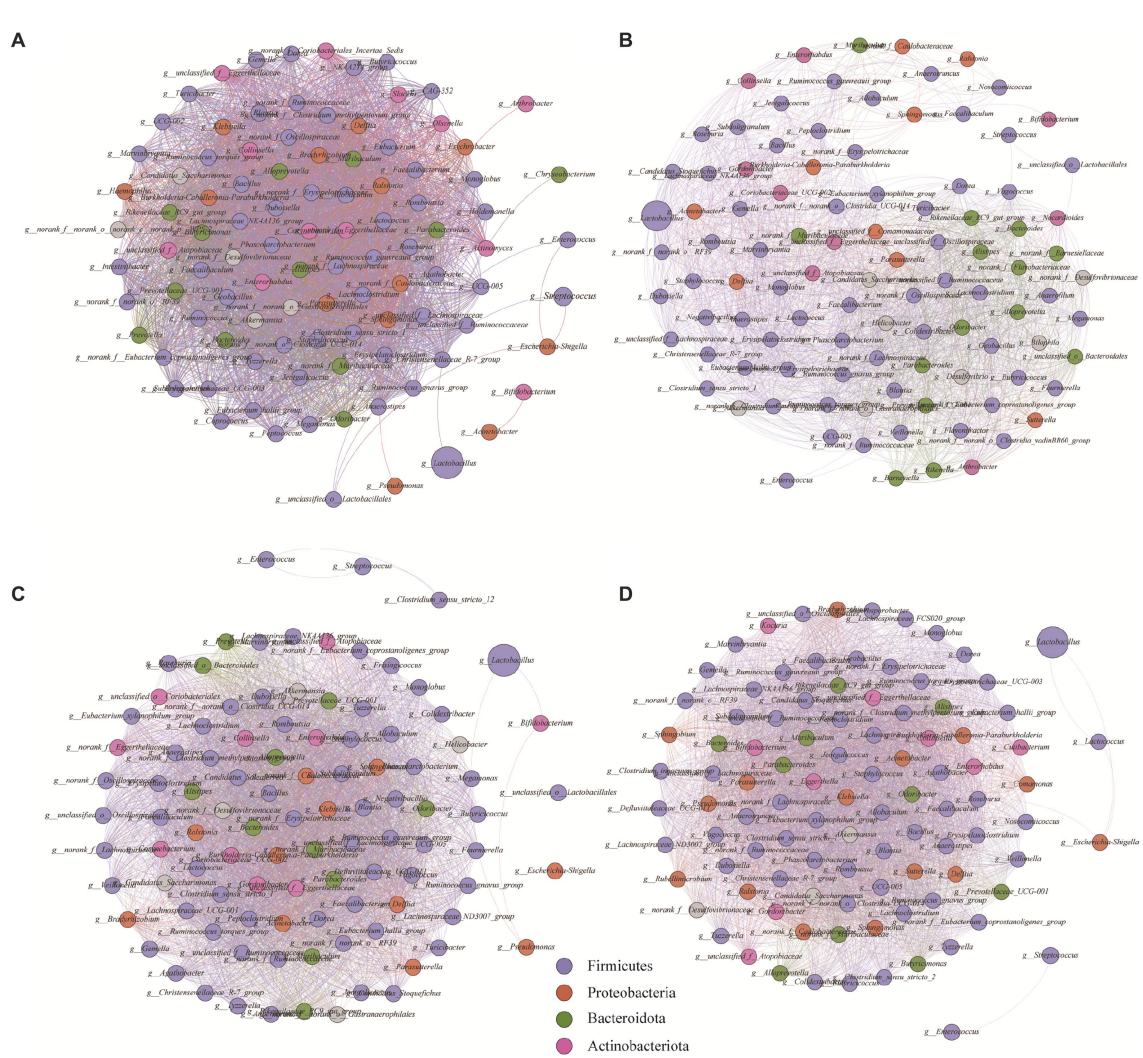
Network analysis of bacterial genera in different treatment groups during fecal fermentation. **(A)** CK; **(B)** NC; **(C)** ULP; **(D)** FLP. The size of the points represents the relative abundance.

The interrelationships among microbial genera (r > 0.95, *p* < 0.05) were analyzed by constructing a network diagram (Figure 5). The number of nodes represents the top 100 genera in each group in terms of relative abundance, and the number of edges represents the level of connection between genera in each treatment group. The CK, NC, ULP, and FLP network diagrams had 3,411, 2,404, 3,669, and 3,857 edges, respectively. The increase in the number of edges in the FLP microbial network indicated that LP intervention led to a more complex microbial network with closer interrelationships among species. Analysis of the average degree of the network revealed that the average degrees of CK (68.22), ULP (72.653), and FLP (77.919) were higher than that of the NC group (48.08), indicating that the degree of intragroup correlation in the NC group was lower than that in the other three groups. Furthermore, there was a clear clustering phenomenon in the ULP and FLP microbial network diagrams, indicating that the microbial network was well-organized and highly ordered (Zheng et al., 2021). Additionally, the addition of LP led to more concentrated microbial network modules with stronger connections between them, suggesting that LP might enhance the complementary effects between dominant groups and weaken the competitive relationships between different groups (Domeignoz-Horta et al., 2020). These results indicate that LP could increase the direct correlations among various bacterial genera, and the relationships between microbes are complex and closely connected, leading to more diverse microbial functions.

### 3.6. Correlation analysis between the core microbiota and metabolites

**Figure 6:**
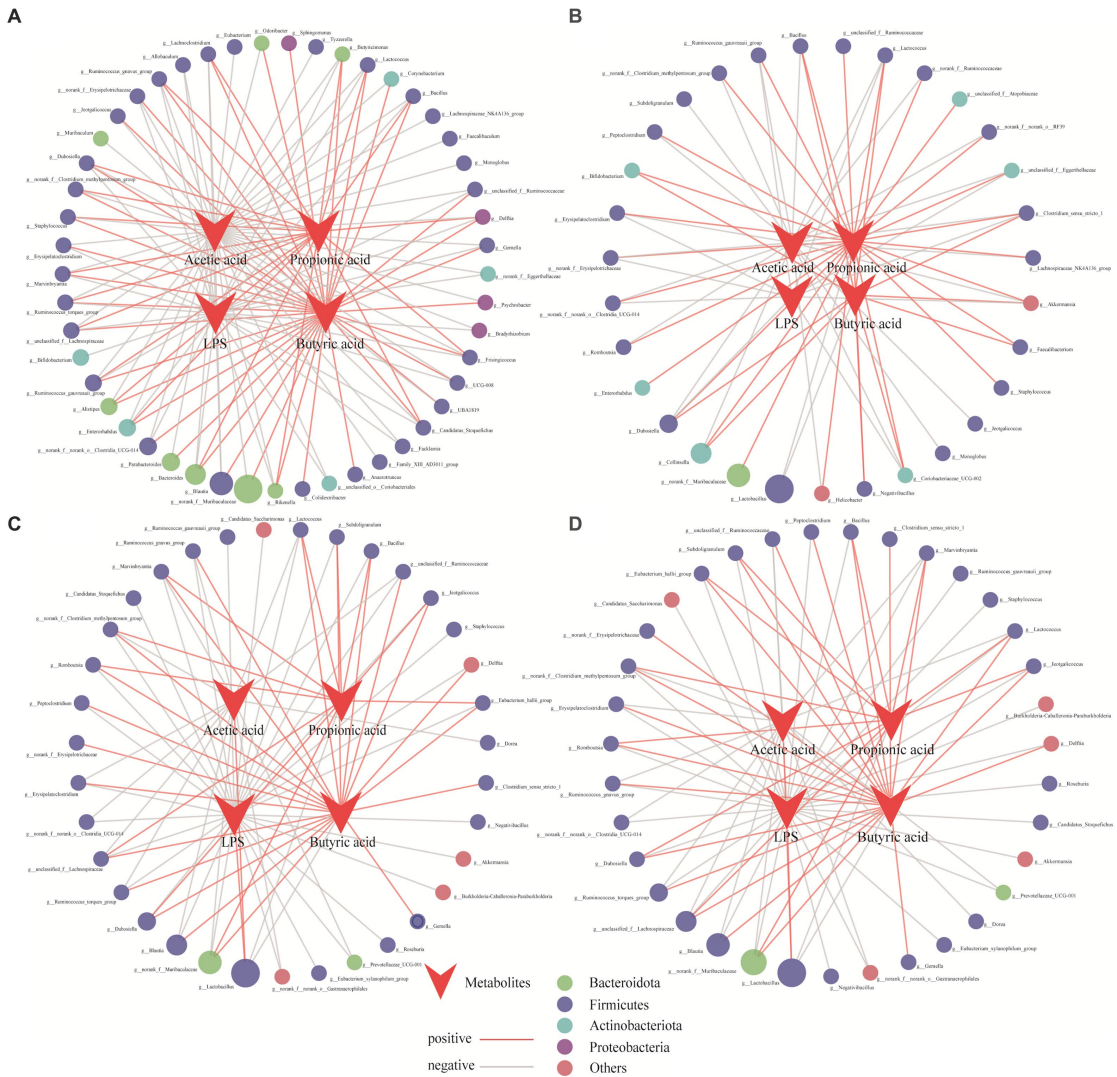
Network analysis of the relationships among acetic acid, propionic acid, butyric acid, LPS, and related bacteria in different treatment groups during fecal fermentation. **(A)** CK; **(B)** NC; **(C)** ULP; **(D)** FLP. The size of the dots represents the relative abundance, while the color represents different phyla. Red and gray represent positive and negative correlations, respectively.

To reveal the effects of the core microbiota on the production of SCFAs and LPS, a collinearity network was constructed to analyze the significant relationships (r > 0.8, *p* < 0.05) among SCFAs, LPS, and the microbiota, and core bacteria were screened. Each node represents a genus involved in SCFA formation. As shown in Figure 6, the core bacterial network diagram of the CK group was more complex than that of the other treatment groups. Compared with the CK group (49, 129), the NC group (31, 71) had fewer nodes and connections. In contrast, the number of nodes and connections in the ULP (35, 80) and FLP (35, 80) groups were closer to that of the CK group, indicating that the addition of LP enhanced the interactions between microbes and metabolites and increased microbial diversity.

In the CK group, 45 core bacterial groups showed a negative correlation with acetic acid. The number of bacterial groups in the NC, ULP, and FLP groups was significantly lower than that in the CK group, which may be related to the metabolic disorders of bacterial groups in T2DM patients. With LP intervention, *Lactobacillus* showed a positive correlation in the ULP and FLP groups, which may be due to the differences in the gut microbiota between normal and diabetic mice and the changes in dominant bacterial species during fermentation. *Lactobacillus* can reduce gut pH, stimulate gut motility, accelerate the excretion of pathogenic bacteria, and inhibit the growth of pathogenic bacteria, such as *Escherichia coli*, *Salmonella*, and *Clostridium*. Therefore, the increase in the number of networks and nodes of *Lactobacillus* in the ULP and FLP groups also confirmed that an increase in acetic acid content in polyphenol-treated groups can alleviate gut microbiota disorders in T2DM patients (Tian et al., 2023a). In addition, compared with the CK group (36), the number of core bacterial genera involved in butyrate synthesis was significantly lower than that in the NC group (11). However, after LP intervention, the number of core bacterial genera involved in butyrate synthesis increased to 22 in both the ULP and FLP groups, leading to an increase in the butyrate content in these groups. Furthermore, the number of core bacterial genera involved in propionate synthesis was reduced by 16 genera in both the ULP and FLP groups (11) compared with the NC group (27), indicating that the addition of LP inhibited the activity of core bacterial genera involved in propionate synthesis. In both the ULP (30) and FLP (30) groups, the number of bacterial genera negatively correlated with LPS was significantly higher than that in the NC group (10). These findings indicate that the addition of LP can effectively reduce the number of LPS-producing bacterial species and reduce damage to the host, which is consistent with the results of Nugent (Nugent et al., 2023).

### 3.7. Corresponding microbial metabolic pathways

**Figure 7:**
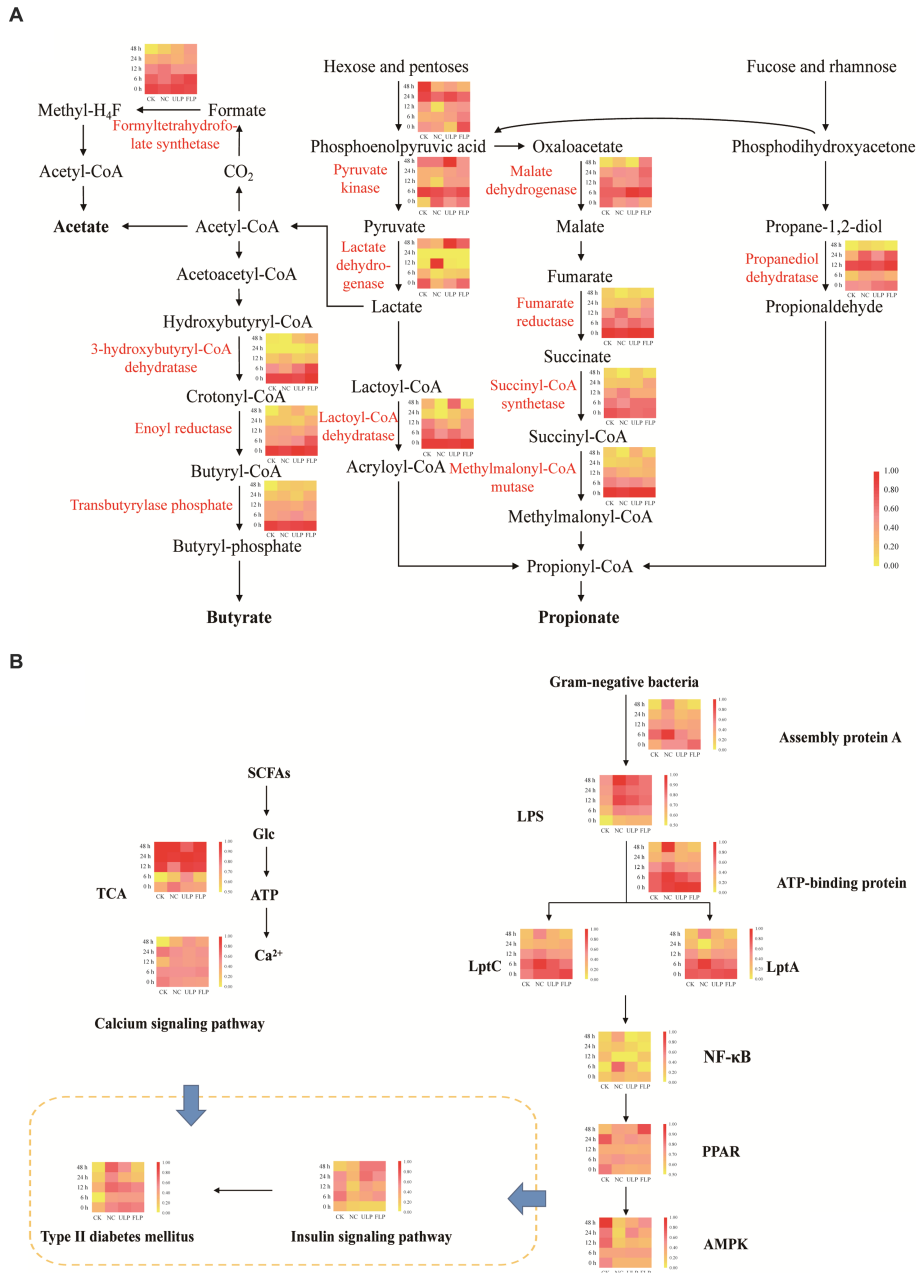
Analysis of polyphenol-mediated microbial metabolic pathways. **(A)** Differential analysis of short-chain fatty acid biosynthesis enzyme pathways in each sample. **(B)** Differential analysis of the mediation of blood glucose levels by polyphenol-regulated microbial metabolic pathways.

Firmicutes can produce acetate and butyrate through direct conversion of acetyl-CoA and the Wood-Ljungdahl pathway (Duncan et al., 2016), while Bacteroidetes can produce propionate through the acrylate pathway, the succinate pathway, and the propanediol pathway (Reichardt et al., 2014). The synthesis pathways of propionate and butyrate are relatively well conserved, with strong substrate specificity, and the synthesis pathways of acetate are more diverse (Ze et al., 2012). In this study, differential analysis was conducted on the key enzymes involved in the biosynthesis pathways of acetic acid, propionic acid, and butyric acid (Figure 7).

There were significant differences in enzyme abundance between the ULP and FLP groups and the NC group (Figure 7A). The enzyme abundances of propanediol dehydratase, succinyl-CoA synthetase, pyruvate kinase, 3-hydroxybutyryl-CoA dehydratase, enoyl reductase, methylmalonyl-CoA mutase, and lactoyl-CoA dehydratase in the FLP group were higher than those in the ULP group before 24 h, indicating that these enzymes are involved in the synthesis of acetate, propionate, and butyrate.

The calcium signaling pathway and insulin signaling pathway are two key pathways for the prevention and alleviation of T2DM. The differential analysis of these pathways (Figure 7B) indicated that glucose metabolism and Ca^2+^ ATP signaling capacity were higher in ULP, suggesting that LP can stimulate the gut microbiota to metabolize carbohydrates to produce SCFAs, increase glucose tolerance by metabolizing glucose, and increase the amount of calcium ions in the cytoplasm to promote insulin release *in vitro*. Additionally, compared to that in NC, the abundances of lipopolysaccharide assembly protein A, ATP-binding cassette transporters, export proteins LptA and LptC, and the NF-κB signaling pathway were significantly reduced in ULP and FLP, while the PPAR signaling pathway and AMPK signaling pathway were significantly upregulated. This indicates that LP inhibited the proliferation of gram-negative bacteria, reduced the secretion of lipopolysaccharides, and promoted insulin receptor phosphorylation. LP can improve glucose tolerance, eliminate insulin resistance, and prevent and alleviate T2DM through these two pathways.

## 4. Conclusion

The gut microbiota is the most complex microbial ecosystem in the body and closely related to T2DM. In this study, LP regulated the concentration of SCFAs, particularly favoring a trend toward recovery to the CK group in the experimental group. Furthermore, LP reversed the dysbiosis of the gut microbiota caused by T2DM, as evidenced by an increase in the abundance of bacterial genera such as *Lactobacillus*, *Blautia*, and *Bacteroides* and a decrease in the abundance of bacterial genera such as *Escherichia-Shigella* and *Streptococcus*. Similarly, after LP intervention, the relationships among microbial species became more complex and interconnected and the correlation between the gut microbiota and metabolites was established through correlation analysis. FLP exhibited superior performance compared to ULP. In summary, this study confirms that LP can improve the gut microbiota and provides a new target for the application of LP in the prevention of T2DM. Moreover, the results obtained in this study confirm the high medicinal potential of LP as a new plant resource. To evaluate its subsequent inhibitory effect on T2DM, further *in vivo* studies are needed.

## Data availability statement

The data presented in the study are deposited in the China National Microbiology Data Center (NMDC) repository, accession number NMDC40043752-NMDC40043811.

## Author contributions

XC: data curation, writing – original draft, writing – review and editing. XW: formal analysis. YR, YS, and ZY: investigation. JG: methodology, conceptualization, and supervision. WP: supervision and resources. All authors contributed to the article and approved the submitted version.
