# Supplementary material for: Lonicera caerulea L. polyphenols improve short-chain fatty acid levels by reshaping the microbial structure of fermented feces in vitro
Source: Front Microbiol. 2023 Oct 27;14:1228700. doi: 10.3389/fmicb.2023.1228700 (PMC10641692; doi:10.3389/fmicb.2023.1228700)
Supplement: Supplementary file 1 [file Data_Sheet_1.docx]

***Lonicera caerulea* L. polyphenols improve short-chain fatty acids by reshaping the microbial structure of fermented faeces *in vitro***

**Xinbo Cao^1^, Xuemeng Wang^1^, Yanxin Ren^1^, Yangcun Sun^1^, Zhichao Yang^1^, Jingping Ge^1, 2 *^, Wenxiang Ping^1, 2 *^**

^1^Engineering Research Center of Agricultural Microbiology Technology, Ministry of Education & Heilongjiang Provincial Key Laboratory of Plant Genetic Engineering and Biological Fermentation Engineering for Cold Region & Key Laboratory of Microbiology, College of Heilongjiang Province & School of Life Sciences, Heilongjiang University, Harbin 150080, China

^2^Hebei University of Environmental Engineering, Hebei Key Laboratory of Agroecological Safety, Qinhuangdao 066102, China

*** Correspondence:**

Email: gejingping@126.com; wenxiangp@aliyun.com

Phone number/Fax: +86-0451-86609106


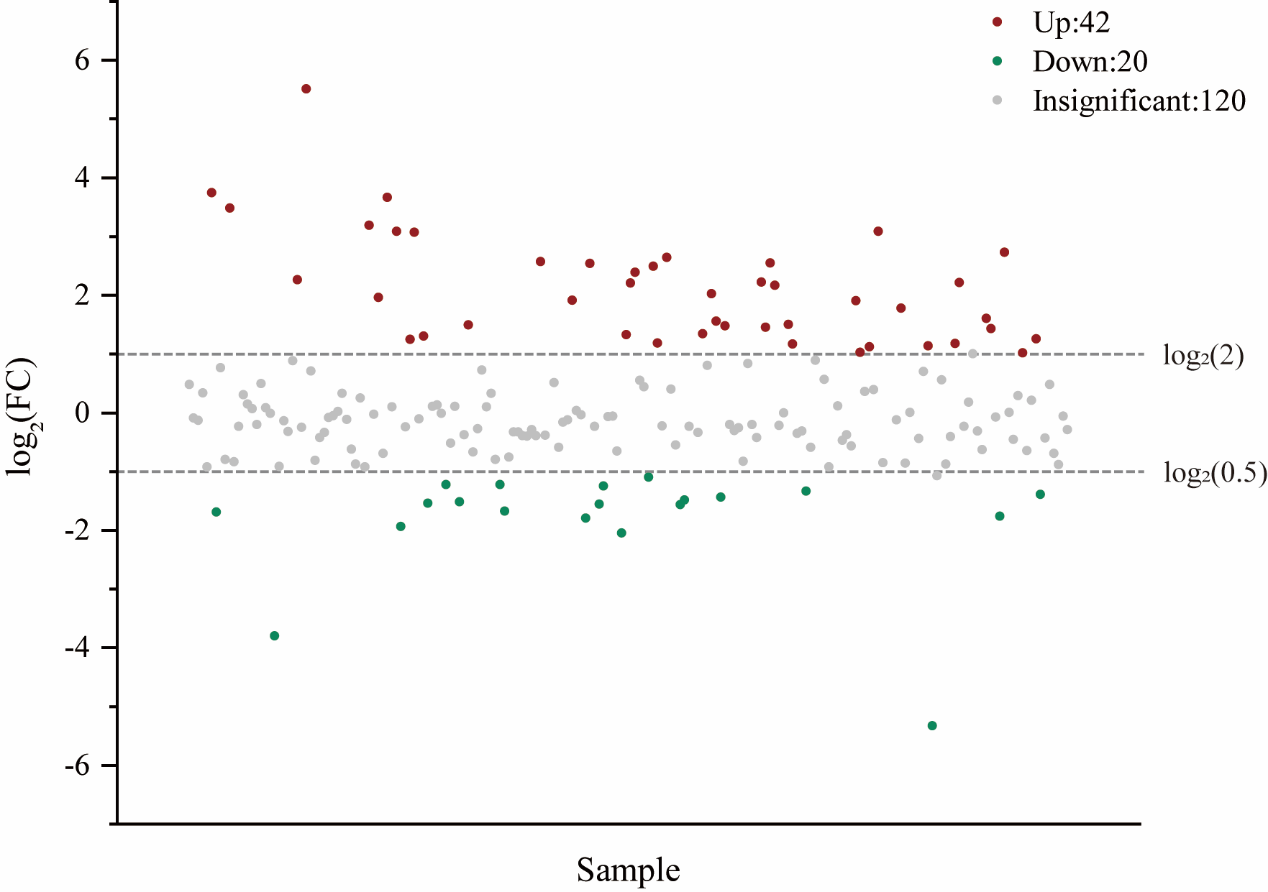


Figure S1. Scatter Plots of screening differential polyphenolic metabolites

of the pre-and post-fermentation

Based on the statistical results, differential polyphenolic metabolites in the ULP and FLP groups were further screened using the fold change (FC) values combined with statistical variable analysis. A fold change of FC ≥ 2 or FC ≤ 0.5 before and after fermentation was considered as significant difference. A scatter plot of differential polyphenolic metabolites was generated with log_2_(FC) as the vertical axis. As shown in Figure S1, a total of 182 polyphenols and their derivatives were detected in the ULP and FLP groups, of which 85 polyphenol metabolites in the FLP group were up-regulated compared with those in the ULP group (42 metabolites significantly different), and 97 metabolites were down-regulated (20 metabolites significantly different).
